# Metabolic Reprogramming of Brain Microglia: Implications for Aging and Aging‐Associated Neurodegenerative Diseases

**DOI:** 10.1111/acel.70660

**Published:** 2026-08-10

**Authors:** Seokjo Kang, Helen S. Goodridge

**Affiliations:** ^1^ Board of Governors Regenerative Medicine Institute Cedars‐Sinai Medical Center Los Angeles California USA; ^2^ Research Division of Immunology in the Department of Biomedical Sciences Cedars‐Sinai Medical Center Los Angeles California USA

**Keywords:** Alzheimer's disease, brain aging, metabolism, microglia, neuroinflammation, Parkinson's disease, sex differences

## Abstract

Microglia, the resident macrophages of the central nervous system (CNS), are key players in maintaining brain and spinal cord homeostasis and protecting the CNS from damage and disease. During aging, the brain undergoes profound changes—including chronic low‐grade inflammation, synaptic dysfunction, and increased vulnerability to neurodegenerative diseases—all of which are closely related to alterations in microglial function. One emerging theme is that microglial metabolism is a crucial determinant of their immune and homeostatic activity. In this mini‐review, we explore how metabolic programs shape brain microglial behavior and how these processes change during aging and in neurodegenerative diseases. We first highlight the link between specific metabolic pathways and key microglial functions, including phagocytosis, cytokine production, and the oxidative stress response. We then discuss how microglial metabolism is reprogrammed during healthy aging and in Alzheimer's disease and Parkinson's disease, including sex‐specific differences. Finally, we examine regulators that influence microglial metabolic states and discuss how these pathways contribute to disease susceptibility and progression. Collectively, recent findings highlight the central role of metabolic reprogramming in shaping microglial responses during aging and in neurodegenerative diseases. We emphasize the need for integrative studies that consider microglial subsets, sex differences, disease context, and upstream molecular regulators to better understand how microglial metabolism contributes to brain health and pathology. A deeper understanding of these pathways may offer new opportunities for therapeutic strategies aimed at restoring microglial homeostasis and mitigating harmful neuroinflammatory processes.

## Graphical abstract

Microglia, the brain's resident macrophages, undergo metabolic reprogramming during aging, which impacts brain health and neurodegenerative disease risk. Understanding mechanisms of microglial metabolic regulation is key to the development of therapeutic strategies to promote homeostasis and mitigate harmful neuroinflammatory processes that contribute to the development of neurodegenerative diseases.

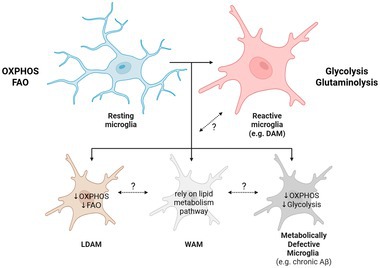

## 1 Introduction

### 1.1 Origins, Subsets, and Roles of Microglia

Microglia are the resident macrophages of the central nervous system (CNS), originating from yolk sac progenitors that colonize the brain during early embryogenesis (Ginhoux et al. 2010). Microglia are long‐lived, self‐renewing cells that are shaped by their CNS environment to maintain a specialized molecular identity (Prinz et al. 2019). In healthy brain tissue, they play essential roles in synaptic pruning, debris clearance, neurogenesis, and immune surveillance (Salter and Stevens 2017; Li and Barres 2018).

Microglia are not a homogeneous population—they exhibit transcriptional diversity across developmental stages, anatomical regions, and disease states. Homeostatic microglia express *Tmem119*, *P2ry12*, and *Sall1* (Butovsky et al. 2014), whereas context‐specific subsets emerge in response to environmental stressors. For instance, disease‐associated microglia (DAM), originally identified surrounding amyloid plaques in Alzheimer's Disease (AD), express *Trem2*, *Apoe* and other genes that are involved in lipid metabolism and phagocytosis (Keren‐Shaul et al. 2017). Functionally, DAM are enriched at sites of pathology where they engulf amyloid‐β, apoptotic cells, and lipid debris, contributing to initial containment of damage; however, their phagocytic efficiency may diminish with disease progression (Keren‐Shaul et al. 2017; Krasemann et al. 2017). DAM‐like states have also been observed in aging and other neurodegenerative diseases, which suggests that this transcriptional program represents a common microglial response to chronic stress and protein aggregation. White matter‐associated microglia (WAM) found in aging white matter upregulate genes involved in lipid handling and phagocytosis, reflecting their role in clearing myelin debris, and their formation depends on TREM2 but not APOE (Safaiyan et al. 2021). Other subsets include interferon‐responsive microglia (IRM), which express antiviral signatures (Sala Frigerio et al. 2019), and lipid‐droplet‐accumulating microglia (LDAM), which display a dysfunctional phenotype with impaired clearance and elevated oxidative stress (Marschallinger et al. 2020). These subsets reflect the functional plasticity of microglia in response to aging and disease, although it is unclear whether they are equivalent across disease states and stages, and how much the activity of each subset changes over time and in response to microenvironmental cues.

### 1.2 Neuroinflammation During Healthy Brain Aging and in Aging‐Associated Neurodegenerative Diseases

Aging is associated with widespread molecular and structural changes in the brain, including chronic low‐grade inflammation, oxidative stress, proteostatic decline, and synaptic dysfunction (López‐Otín et al. 2013; Mattson and Arumugam 2018). Even in the absence of overt pathology, aged brains exhibit increased expression of inflammatory cytokines, elevated microglial activation, and a sensitized neuroimmune environment (Mosher and Wyss‐Coray 2014). This chronic inflammatory milieu is thought to sensitize the brain to secondary insults and lower the threshold for disease onset. In parallel, increased inflammatory mediator signaling across the blood–brain barrier (BBB) during aging amplifies neuroinflammation (Yousef et al. 2019). In neurodegenerative diseases such as AD and Parkinson's Disease (PD), the inflammatory response is further exacerbated by the infiltration of peripheral immune cells into the CNS due to BBB damage (Sweeney et al. 2018; Suk 2026), and this contributes to neuronal loss, synaptic damage, and progressive cognitive impairment (Heneka et al. 2015; Subramaniam and Federoff 2017). The resulting self‐perpetuating cycle of neuroinflammation may prime microglia toward maladaptive states and create a permissive environment for disease progression.

### 1.3 Functional Changes in Microglia Responding to Stress

With aging, microglia shift from a homeostatic to a primed or reactive state. Aged microglia exhibit elevated basal expression of pro‐inflammatory mediators (*Il1b*, *Tnf*, *Il6*) and exaggerated responses to immune stimuli (Norden and Godbout 2013). Their phagocytic efficiency declines, impairing clearance of apoptotic cells and protein aggregates such as amyloid‐β (Aprahamian et al. 2008; Marschallinger et al. 2020). In addition to this impaired clearance, microglia may also become more active in synaptic pruning, particularly in aging and AD. Complement proteins such as C1q and C3 mark synapses for elimination, and microglia remove these tagged synapses (perhaps sometimes inappropriately), leading to synaptic loss and cognitive decline (Hong et al. 2016; Shi et al. 2017).

Furthermore, aged microglia exhibit dysregulated interactions with neurons and astrocytes, contributing to neuroinflammatory feedback loops and impaired support of synaptic and vascular integrity (Colonna and Butovsky 2017; Liddelow et al. 2017; Clarke et al. 2018). These functional changes reflect a loss of microglial flexibility and contribute to the transition from protective to detrimental roles in aging and aging‐associated neurodegenerative diseases. Elucidating the mechanisms behind these shifts, particularly the role of metabolic reprogramming, may offer novel strategies for restoring microglial homeostasis.

## 2 Microglial Metabolism as a Functional Determinant

### 2.1 Metabolic Pathways and Their Functional Integration

Microglia rely on multiple interconnected metabolic pathways to meet their energetic and biosynthetic demands, including glycolysis, oxidative phosphorylation (OXPHOS), fatty acid oxidation (FAO), and glutamine metabolism (glutaminolysis). In their homeostatic state, microglia primarily depend on OXPHOS, where glucose‐derived pyruvate enters the tricarboxylic acid (TCA) cycle and fuels the electron transport chain (Sokolov et al. 2015), supporting surveillance and synaptic maintenance (Bernier, York, Kamyabi, et al. 2020). Upon activation by inflammatory stimuli such as lipopolysaccharide (LPS), amyloid‐β, or α‐synuclein, microglia undergo a shift toward aerobic glycolysis, or the “Warburg effect” (Rubio‐Araiz et al. 2018; Qiao et al. 2019). Although glycolysis is less efficient in ATP yield, it provides rapid energy and supplies intermediates for nucleotide and lipid synthesis. Its metabolites can enter the pentose phosphate pathway, generating NADPH that supports anabolic processes and fuels production of reactive oxygen species (ROS) and nitric oxide for immune defense (Tu et al. 2019). FAO contributes to microglial plasticity by generating acetyl‐CoA for mitochondrial respiration and anti‐inflammatory programs, but impaired FAO in aging or neurodegenerative diseases promotes lipid droplet accumulation and dysfunctional inflammatory states (Marschallinger et al. 2020; Yang et al. 2021). Glutaminolysis also plays a critical role by converting glutamine into α‐ketoglutarate to replenish the TCA cycle. Beyond serving as a metabolic intermediate, α‐ketoglutarate can modulate the epigenetic landscape of microglia, promoting phagocytic gene programs and enhancing amyloid‐β clearance (McManus et al. 2025).

Together, these pathways act as metabolic switches that determine whether microglia adopt homeostatic, inflammatory, or reparative states. When intact, they enable efficient surveillance, phagocytosis, and trophic support; but when disrupted by mitochondrial dysfunction, impaired lipid handling, or altered amino acid metabolism, microglia shift toward maladaptive phenotypes characterized by chronic inflammation, oxidative stress, and defective clearance (Baik et al. 2019; Afridi et al. 2020; Bernier, York, and MacVicar 2020; Sadeghdoust et al. 2024).

### 2.2 Metabolic Features of Microglial Subsets

**FIGURE 1:**
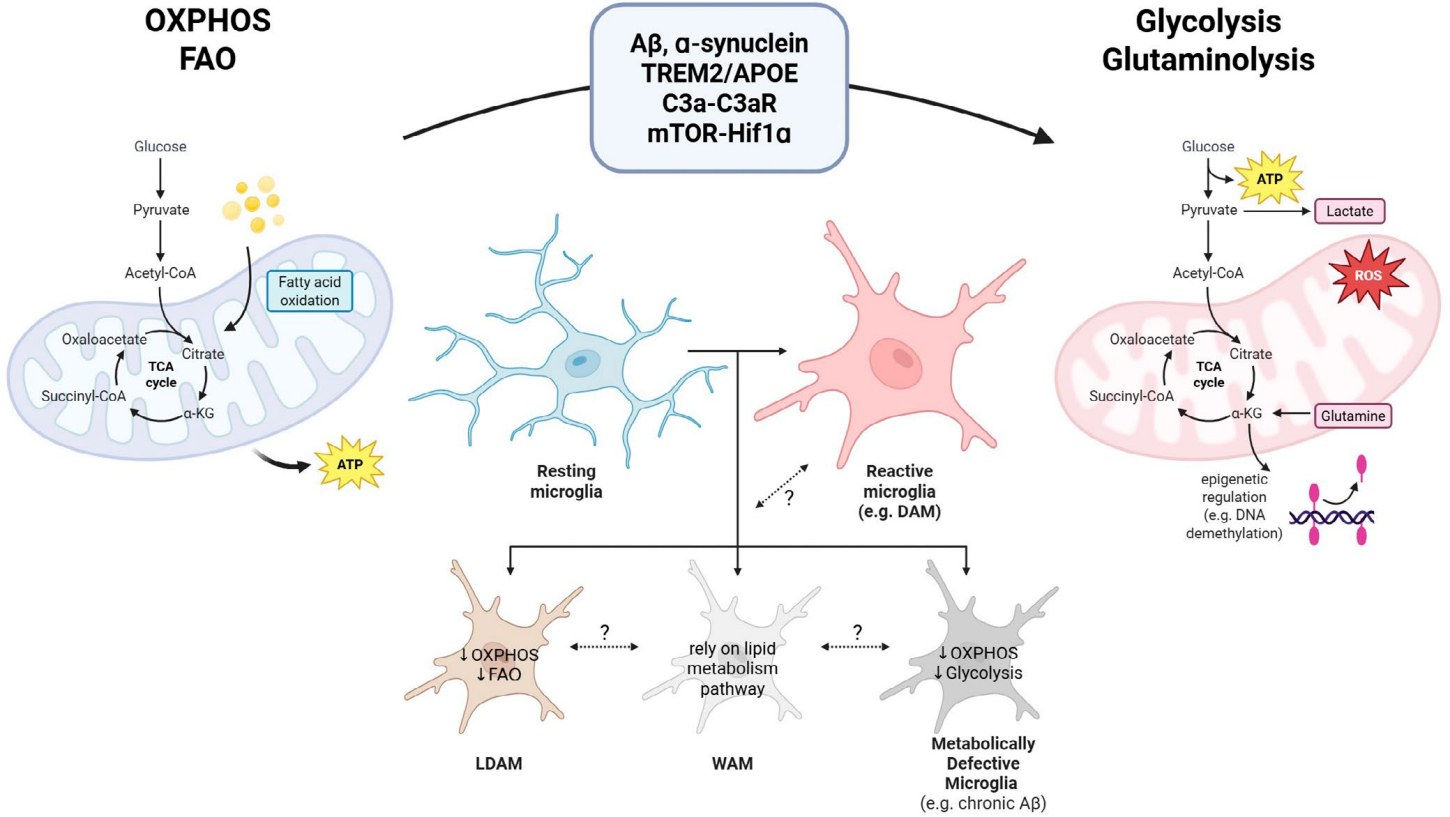
Microglial metabolic pathways and functional states. Schematic representation of major metabolic pathways that shape microglial function (created using BioRender.com). In their homeostatic state, microglia rely on oxidative phosphorylation (OXPHOS) and fatty acid oxidation (FAO) to support energy‐efficient surveillance and phagocytosis. Upon activation by stimuli such as amyloid‐β (Aβ) or α‐synuclein, microglia shift toward glycolysis, enabling rapid ATP production, cytokine release, and reactive oxygen species (ROS) generation. Glutaminolysis replenishes the tricarboxylic acid (TCA) cycle and contributes to epigenetic regulation of phagocytic and inflammatory programs. Key regulators, including TREM2/APOE (lipid handling), C3a‐C3aR, and mTOR‐Hif1ɑ (glycolytic reprogramming), determine whether microglia adopt homeostatic, inflammatory, or reparative states. Subset‐specific metabolic features are indicated: Disease‐associated microglia (DAM; enhanced glycolysis), lipid‐droplet‐accumulating microglia (LDAM; impaired OXPHOS and FAO), white‐matter‐associated microglia (WAM; reliance on lipid metabolism pathway for myelin debris clearance), and metabolically defective microglia (impaired OXPHOS and glycolysis following chronic Aβ exposure).

Distinct microglial subsets display characteristic metabolic programs that reflect their specialized roles in health and disease (Figure 1). For example, DAM, which emerge around amyloid plaques in AD, show enhanced glycolysis and lipid catabolism to sustain energy‐intensive phagocytosis, while downregulating homeostatic and surveillance genes (Keren‐Shaul et al. 2017; Kang et al. 2024). In contrast, LDAM in the aging brain exhibit defective fatty acid oxidation, mitochondrial dysfunction, and elevated oxidative stress, leading to impaired clearance capacity and proinflammatory activity (Marschallinger et al. 2020). IRM, which are defined by type I interferon‐stimulated genes, remain poorly characterized metabolically. Although direct evidence is lacking, studies in macrophages during *Mycobacterium tuberculosis* infection show that type I interferon reduces glycolysis and induces mitochondrial stress (Sala Frigerio et al. 2019; Olson et al. 2021). Moreover, recent work has implicated IRF5 as a regulator of microglial lipid metabolism, linking interferon signaling to pathways controlling myelin clearance and cholesterol handling (Montilla et al. 2025). WAM in aging white matter depend on TREM2 signaling to clear myelin debris, suggesting a reliance on lipid handling pathways (Safaiyan et al. 2021). Although the role of glutamine metabolism in these subsets is less well defined, emerging evidence indicates that glutaminolysis may sustain inflammatory activation and phagocytic programming under disease conditions (Zhang et al. 2024; McManus et al. 2025). Collectively, these examples illustrate how metabolic specialization is a defining feature of microglial heterogeneity across different contexts of aging and neurodegenerative diseases.

## 3 Context‐Specific Changes in Microglial Metabolism

### 3.1 Healthy Aging

During aging, microglia progressively lose metabolic efficiency (Table 1). Mitochondrial dysfunction reduces OXPHOS and ATP production while increasing ROS, shifting microglia toward glycolysis, which is a faster but less sustainable pathway that amplifies inflammatory tone (Geng et al. 2020; Kang et al. 2024). This glycolytic reprogramming is reflected in the emergence of DAM, which display enhanced glycolysis and lipid metabolism and are increasingly detected in aged brains even in the absence of overt pathology (Geng et al. 2020; Kang et al. 2024). A subset of aged microglia also accumulates intracellular lipid droplets, forming LDAM. These cells display defective FAO, impaired clearance of apoptotic cells and proteins, and heightened secretion of pro‐inflammatory mediators (Marschallinger et al. 2020). Together, these changes suggest that microglia are collectively protective early in life, but with age become less resilient and more inflammatory. Whether this reflects population‐level reprogramming or progressive exhaustion of individual cells remains unclear.

**TABLE 1:** Context‐specific microglial metabolic changes in aging and neurodegenerative diseases.

Context	Metabolic features	Functional consequences	Key regulators	References
Healthy aging	↓ OXPHOS, ↑ glycolysis, ↑ ROS, lipid droplet accumulation (LDAM), impaired FAO	Chronic low‐grade inflammation, impaired clearance of apoptotic cells/proteins, metabolic priming	AKT–mTOR–HIF1α, C3a–C3aR	Geng et al. (2020), Kang et al. (2024), Marschallinger et al. (2020)
Alzheimer's disease (AD)	Enhanced glycolysis, impaired mitochondrial respiration, ↑ glutaminolysis	Inefficient Aβ clearance, inflammasome activation (NLRP3), chronic inflammation, synaptic loss	Aβ, TREM2–APOE axis, mTOR–HIF1α	Baik et al. (2019), Ulland et al. (2017), Fairley et al. (2021), Zhang et al. (2024), McManus et al. (2025)
Parkinson's disease (PD)	PKM2‐dependent glycolysis, impaired OXPHOS, lipid droplet accumulation, FAO impairment	Chronic cytokine production, oxidative stress, reduced clearance, dopaminergic neuron vulnerability	α‐synuclein, TBK1–ACSL1	Qiao et al. (2019), Zhang et al. (2025), Han et al. (2025)
Sex differences	Females: stronger glycolytic bias, DAM enrichment, enhanced senescence	Female‐biased inflammation, greater plaque load, accelerated AD susceptibility	PFKFB3, C3a–C3aR, estrogen signaling	Kang et al. (2024), Ocañas et al. (2023), Guillot‐Sestier et al. (2021), Cleland et al. (2024)
	Males: relatively small increase in glycolysis			

### 3.2 Alzheimer's Disease and Parkinson's Disease

Neurodegenerative diseases exacerbate microglial metabolic stress (Table 1). In AD, exposure to amyloid‐β drives a metabolic shift from OXPHOS to glycolysis via the mTOR–HIF‐1α pathway, impairing energy metabolism, phagocytosis, and cytokine responses (Baik et al. 2019). This shift characterizes DAM, which emerge around amyloid plaques and display enhanced glycolysis and lipid metabolism with downregulated homeostatic genes (Keren‐Shaul et al. 2017). DAM are thought to initially promote amyloid clearance and plaque compaction, but they may become less efficient and proinflammatory with chronic activation, highlighting the need for longitudinal studies to define how microglial states evolve across disease progression (Ulland et al. 2017; Fairley et al. 2021). In addition, LDAM and IRM have been detected in AD brains, suggesting that multiple metabolically altered microglial states coexist within the disease milieu (Sala Frigerio et al. 2019; Marschallinger et al. 2020). However, how these single‐cell‐defined subsets correspond to metabolic and inflammatory changes reported in earlier bulk‐tissue studies remains unclear, underscoring the need for integrated analyses combining single‐cell profiling with functional assays.

Mitochondrial dysfunction emerges as a central hallmark of microglial immunometabolism in AD, where reduced OXPHOS capacity drives chronic inflammation and loss of homeostatic functions (Fairley et al. 2021). Emerging evidence further implicates glutamine metabolism in inflammatory activation, with glutaminolysis promoting NLRP3 inflammasome activity and maladaptive cytokine release (Zhang et al. 2024; McManus et al. 2025). In PD, α‐synuclein aggregates induce PKM2‐dependent glycolysis and impair OXPHOS, resulting in chronic cytokine production and dopaminergic neuron vulnerability (Qiao et al. 2019; Zhang et al. 2025). In parallel, altered lipid metabolism, including microglial lipid droplet accumulation mediated by ACSL1 induction downstream of TBK1 and FAO impairment, adds oxidative stress and further disrupts microglial clearance (Han et al. 2025). Collectively, these findings highlight that in both AD and PD, disease‐specific stressors reprogram microglia toward glycolysis and lipid dysregulation, establishing maladaptive inflammatory states that accelerate neurodegeneration. The increasing resolution of single‐cell and spatial approaches will be crucial to link these defined microglial subsets with the broader metabolic alterations long observed in bulk analyses.

### 3.3 Sex Differences in Microglial Metabolism

Sex differences are notable in aging‐associated neurological disorders, and both genetic factors and hormonal changes have been implicated (Lopez‐Lee et al. 2024; Bonkhoff et al. 2025). Sex strongly shapes microglial metabolism during aging and in aging‐associated neurodegenerative diseases (Table 1). Transcriptomic and metabolic profiling revealed that aged female microglia undergo stronger glycolytic reprogramming than males, driven by AKT–mTOR–HIF1α signaling and reinforced by autocrine C3a–C3aR pathways, resulting in a greater abundance of DAM (Kang et al. 2024). Complementing this, female hippocampal microglia show heightened inflammatory activation with age, predisposing to neuroinflammation and AD vulnerability (Ocañas et al. 2023).

Consistent with this, in the APP/PS1 mouse model of AD, female microglia exhibit a pronounced shift toward glycolysis and upregulation of glycolytic enzymes such as PFKFB3—changes that are not observed to the same degree in males (Guillot‐Sestier et al. 2021). These metabolic alterations are mirrored in human post‐mortem AD tissue, where female patients show higher amyloid plaque load and distinct microglial morphology compared with males (Guillot‐Sestier et al. 2021). In parallel, estrogen modulates these programs by dampening DAM‐related genes (*Trem2*, *Apoe*, *Lpl*) and influencing glycolysis and lipid metabolism, partially buffering female‐biased inflammatory responses (Cleland et al. 2024).

Together, these findings converge on the idea that female microglia are more prone to glycolytic bias, DAM enrichment, and inflammation‐associated dysfunction in brain aging and AD, underscoring sex as a critical variable in microglial metabolism and disease susceptibility.

## 4 Regulation of Microglial Metabolism

### 4.1 Genetic Variants Regulating Microglial Metabolism

Genetic risk factors for neurodegeneration often converge on metabolic regulation in microglia. TREM2, a lipid‐sensing receptor, is indispensable for the transition to DAM by regulating microglial glycolysis (Wang et al. 2025). Loss‐of‐function mutations impair lipid uptake and mitochondrial respiration, preventing the DAM shift and reducing plaque compaction in both mouse AD models and in AD patients (Wang et al. 2015; Keren‐Shaul et al. 2017; Ulland et al. 2017). Variants of APOE, which regulates cholesterol transport and lipid handling, are the strongest genetic risk factor for late‐onset AD. The APOE4 isoform alters microglial lipid metabolism, enhances glycolytic bias, and promotes mitochondrial stress, thereby exacerbating pro‐inflammatory activation and impairing phagocytic efficiency (Shi and Holtzman 2018; Fernandez et al. 2019; Lee et al. 2023). Recent evidence further suggests that these APOE4‐driven metabolic disturbances may be more pronounced in females, potentially contributing to sex‐specific vulnerability to AD (Arnold et al. 2020).

### 4.2 Signaling Modulators of Microglial Metabolism

Beyond genetic variants, intracellular signaling pathways tightly regulate metabolic states. mTOR functions as a metabolic checkpoint, promoting glycolysis through HIF‐1α activation while repressing autophagy and OXPHOS; its hyperactivation in AD skews microglia toward pro‐inflammatory states (Baik et al. 2019). Complement signaling also feeds directly into metabolism. C3a/C3aR autocrine loops in aging/AD drive a glycolytic, DAM‐like program that links innate immune tone to metabolic reprogramming, especially in females (Kang et al. 2024). Additional regulators include AMPK, which counterbalances mTOR by promoting mitochondrial biogenesis, fatty acid oxidation, and autophagy while suppressing pro‐inflammatory signaling (Saito et al. 2019). PPARγ, a lipid‐sensing transcription factor, also supports anti‐inflammatory metabolism and enhances microglial phagocytosis, promoting mitochondrial respiration and lipid catabolism via increased OXPHOS and FAO (Fumagalli et al. 2018).

Together, these findings highlight how genetic risk factors and signaling pathways converge on microglial metabolism to determine whether microglia adopt homeostatic, inflammatory, or reparative phenotypes.

## 5 Future Directions in Microglial Metabolism

Despite recent progress, our understanding of microglial metabolism remains incomplete. Future research will benefit from integrated multi‐omic approaches combining transcriptomic, epigenomic, metabolomic, and proteomic profiling at spatial and single‐cell resolution to capture how microglial states are remodeled across aging and neurodegenerative diseases (Ayata et al. 2018; Masuda et al. 2020; Engskog‐Vlachos et al. 2025; Zhou and Glass 2025). Such approaches are critical for disentangling how intrinsic factors (e.g., sex chromosome effects, genetic variants) and extrinsic cues (e.g., sex steroid hormones, inflammatory milieu, metabolites) converge on metabolic programs. Sex‐specific regulation of microglial metabolism represents a key area of focus, as female‐biased glycolytic and inflammatory phenotypes may underlie greater vulnerability to AD (Guillot‐Sestier et al. 2021; Hou et al. 2024; Kang et al. 2024). Addressing this dimension is essential for both mechanistic insight and precision medicine.

Beyond the brain, similar metabolic transcriptional reprogramming has been observed in spinal cord microglia during amyotrophic lateral sclerosis (ALS). DAM‐like signatures have been identified in ALS models and human tissue (Krasemann et al. 2017; Jauregui et al. 2023; Shen et al. 2024), suggesting that metabolic dysfunction and lipid dysregulation may be shared mechanisms driving neuroinflammation across neurodegenerative diseases. Elucidating how microglial metabolism adapts within the spinal cord environment will be critical for understanding disease progression and therapeutic responses in ALS. Microglial metabolism is also increasingly implicated in neuroimmunological diseases such as multiple sclerosis, with lesion‐associated microglia expressing DAM‐ or MGnD‐like gene programs, including *Apoe*, *Spp1*, and *Itgax* (Krasemann et al. 2017; Masuda et al. 2019).

More broadly, studies comparing the metabolic and functional properties of these microglial states in distinct disease contexts (different diseases or disease stages) (Sadeghdoust et al. 2024), as well as across the lifespan in healthy individuals, could shed light on their roles in neuroprotection versus neuropathology and may identify common mechanisms of neuroinflammation that can be targeted therapeutically in diverse diseases. For instance, microglia are active in the developing brain during childhood and adolescence as well as in later life, and developmental and aging/disease‐associated microglial subsets exhibit overlapping but also distinct properties (Catale and Garel 2025). Comparing microglia in neurodegenerative diseases that manifest in early life versus middle age or later life could also be informative. It will be important to distinguish between the intrinsic effects of genetic variants, which may be disease‐specific, and broader non‐specific responses to toxic aggregates, neuron damage, and activatory signals from other cell types. For instance, mutations that decrease expression of the mitochondrial protein frataxin in Friedreich's Ataxia (Della Valle et al. 2024; Sciarretta et al. 2024; Pernaci et al. 2026) profoundly impact microglia and are thought to drive development of the disease in early life, whereas many genetic variants may only compound neurogenerative disease risk in later life in combination with other genetic and environmental factors. Microglial roles and responses may also vary depending on the types of neurons and other cell types they interact with.

Another important direction will be to integrate microglial metabolic profiling with emerging imaging and computational approaches that can link cellular states to clinical phenotypes. Advanced computational approaches that facilitate more precise assessment of microglial morphology are now revealing insights into the relationship between microglial morphology and function (Vidal‐Itriago et al. 2022; Green and Rowe 2024). Moreover, molecular imaging tools such as TSPO PET and next‐generation microglia‐specific radiotracers provide in vivo readouts of regional microglial reactivity (Jacobs et al. 2012; Heneka et al. 2025), with studies showing that increased tracer binding correlates with cognitive decline and neuropathological burden in AD (Kreisl et al. 2013; Dani et al. 2018), while potentially exerting stage‐dependent protective effects in prodromal phases (Hamelin et al. 2016; Femminella et al. 2019). Complementary advances in fluorescence lifetime imaging microscopy (FLIM) combined with deep learning‐based morphological classification enable high‐resolution assessment of microglial activation states by integrating NADH metabolic signatures with structural features (Mukherjee et al. 2022). These imaging and computational frameworks offer promising avenues to bridge microglial metabolism with region‐specific vulnerability and cognitive trajectories in neurodegenerative diseases.

Finally, therapeutic opportunities lie in targeting microglial metabolism to restore homeostasis. Interventions that modulate mTOR, AMPK, or PPARγ signaling, or that leverage metabolic–epigenetic crosstalk, hold potential to delay microglial aging, enhance clearance of pathologic proteins, and mitigate neuroinflammation (Fairley et al. 2021; Lepiarz‐Raba et al. 2023, 2024; Codocedo et al. 2024). However, modulating these signaling molecules is complex and likely stage‐dependent. Transient activation may support metabolic adaptation and repair, whereas chronic hyperactivation can suppress autophagy and promote persistent inflammation. A deeper understanding of pathway dynamics across disease stages will be essential for designing safe and effective metabolism‐targeted interventions.

## 6 Limitations and Challenges

As we have discussed, recent studies have revealed important insights into microglial subsets and the link between their metabolic profiles and their functional properties and roles in neuroprotection versus neurodegeneration. Nevertheless, much of our current understanding has been informed by mouse studies, which incompletely recapitulate the cellular diversity, lifespan‐associated changes, and inflammatory environments observed in the human brain, and even in mice, it is currently very difficult to observe and modulate microglial metabolism in vivo. Most studies have therefore evaluated isolated microglia in culture‐based assays outside the physiological context of the brain where their behavior and responsiveness are impacted over time by exposure to other cell types, factors from the blood and cerebrospinal fluid, and other physiological influences. For instance, sex differences evaluated in vitro do not fully recapitulate gonadal hormone effects, including the impact of changes in hormone levels across the lifespan. Moreover, most analyses provide indirect readouts of activation states rather than comprehensive metabolic profiling. Addressing these limitations will require more relevant human model systems, non‐invasive technologies for longitudinal in vivo monitoring in both mice and humans, and multimodal approaches to integrate imaging, single‐cell profiling, and functional analyses.

## 7 Conclusion

Microglial metabolism is emerging as a central determinant of brain aging and neurodegenerative diseases. Across contexts of healthy aging, AD and PD, and sex‐specific trajectories, microglia undergo profound metabolic remodeling that shapes their functional states. Importantly, distinct subsets such as DAM, LDAM, IRM, and WAM exhibit unique metabolic signatures that influence clearance, inflammation, and tissue repair, underscoring the need to study microglial diversity in parallel with their metabolic programs. Genetic risk factors and signaling pathways further reinforce this reprogramming, tipping the balance between protective and maladaptive phenotypes. As emerging imaging and computational tools increasingly allow microglial states to be mapped in vivo and in human tissue, integrating multi‐omic insights with functional studies, the field is now poised to identify therapeutic strategies that restore metabolic homeostasis, rejuvenate microglial function, and mitigate neuroinflammation in age‐related disease.

## Author Contributions

S.K. and H.S.G. both contributed to the conceptualization, writing, and editing of the manuscript.

## Funding

This work was supported by the Alzheimer's Association (ABA‐25‐1372697).

## Conflicts of Interest

The authors declare no conflicts of interest.

## Data Availability

The authors have nothing to report.
